# Serum phospholipids during aging: A comprehensive systematic review of cross-sectional and case-control studies

**DOI:** 10.34172/hpp.025.42914

**Published:** 2025-05-06

**Authors:** Meysam Zarezadeh, Mahsa Mahmoudinezhad, Amir Hossein Faghfouri, Nima Radkhah, Mehrdad Jamali, Parsa Jamilian, Zohreh Ghoreyshi, Alireza Ostadrahimi

**Affiliations:** ^1^Faculty of Nutrition and Food Science, Tabriz University of Medical Sciences, Tabriz, Iran; ^2^Maternal and Childhood Obesity Research Center, Urmia University of Medical Sciences, Urmia, Iran; ^3^School of Medicine, Keele University, Staffordshire, UK

**Keywords:** Aging, Lipidomics, Phosphatidylcholine, Phosphatidylethanolamine, Phosphatidylinositol, Phospholipids

## Abstract

**Background:**

The lipidome, as a subset of metabolomics, can undergo significant variations due to several factors, including the aging process. Therefore, this study aims to summarize the relationship between aging and alterations in plasma phospholipids.

**Methods:**

A comprehensive search was conducted in MEDLINE (PubMed), Scopus, Embase, Web of Science, and Google Scholar databases up to September 2023. The PRISMA guidelines were adhered to throughout all stages of the review process. Cross-sectional and case-control studies that investigated the relationship between aging and phosphatidylcholine (PC), lysophosphatidylcholine (LPC), sphingomyelins (SM), ceramides, phosphatidylethanolamines (PE), and phosphatidylinositol (PI) were included.

**Results:**

A total of 8486 studies were identified, of which 32 met the predefined inclusion criteria. The systematic review included data from 70,499 participants. The findings revealed that four studies reported a positive association between PCs and aging in both sexes, while one study reported an inverse relationship. Additionally, two studies found that PCs were positively associated with aging in men and negatively associated in women. Furthermore, four studies indicated a negative association between PC metabolites and the aging process. With regard to LPCs, two studies demonstrated a positive correlation, and two studies showed an inverse correlation with aging in both sexes. For SMs, five studies reported a positive association, whereas two studies identified an inverse association with aging trends. Similarly, five studies reported a positive correlation between PE levels and aging, while two studies showed a negative correlation.

**Conclusion:**

Phospholipids play a critical role in the aging process, aging-related diseases, and the regulation of lifespan. A reduction in the levels of PCs and LPCs has been identified as a characteristic feature of aging.

## Introduction

 The increase in life expectancy and the growing proportion of aging populations significantly strain healthcare systems in developed countries. Aging impacts all bodily systems and biological processes, from the genetic level to entire organs, resulting in functional changes. It is also a significant risk factor for various conditions, including cancer, ischemic heart disease, Alzheimer’s disease (AD), osteoarthritis, and diabetes.^1-4^ Comprehending the biology of aging is essential for developing long-term strategies to improve the health and well-being of the elderly.

 In recent years, significant interest has focused on the link between metabolomics and aging.^5^ Lipidomics, a subset of metabolomics, focuses on identifying and quantifying human lipids, including a vast array of metabolites.^6^ Lipids serve as both structural and signaling molecules and are associated with age-related diseases such as cardiovascular disease (CVD),^7^ metabolic syndrome,^8^ macular degeneration,^9^ Alzheimer disease,^10^ and at least some types of stroke.^11^ It has been demonstrated that plasma lipidome composition varies significantly with age.^12,13^ For instance, while some phosphoglycerides increase with age, others decrease.^14,15^ Similarly, it has been demonstrated that sphingomyelin (SM) levels vary with age and sex based on the SM species.^13,16^

 It is well known that circulating ceramides are associated with insulin resistance, type 2 diabetes, prediabetes, and obesity.^17-19^ Moreover, circulating ceramides are associated with an increased risk of death from CVD.^20,21^ Long-chain (dihydro) ceramides are positively associated with the risk of type 2 diabetes mellitus and CVD. In contrast, very long-chain ceramides and more complex sphingolipids, such as lactosylceramides, have demonstrated negative associations.^22,23^ Moreover, sphingolipid synthesis enzymes are potential for CVD risk reduction targets,^24^ and inhibition of glycosphingolipid biosynthesis has been shown to reduce atherosclerosis in mice.^25^ Previous research has indicated that men have higher circulating ceramide levels than premenopausal women.^26^ However, postmenopausal women experience a more rapid increase in ceramide levels compared to men.^27^ It has been demonstrated that during healthy aging, SM levels rise in women but fall in men.^16^ Pathological conditions may affect the concentrations of specific lipid metabolites. For example, AD is associated with disruptions in the normal pattern of change in SM levels with aging.^16,28^ Similarly, an increase in acylcarnitine levels is associated with a higher risk of type 2 diabetes.^29^

 These examples suggest that the role of plasma phospholipids in the aging process and age-related diseases has been increasingly studied and lipidomics research could provide important insights into the pathogenesis of age-related chronic diseases. However, findings are inconsistent, particularly regarding the impact of different phospholipid classes (e.g., phosphatidylcholines [PCs], lysophosphatidylcholines [LPCs], sphingomyelins [SMs], and ceramides) across sexes, body mass index (BMI) categories, and tissues. Additionally, previous studies often lack a comprehensive comparison of phospholipid levels in aging populations stratified by gender and health conditions. This systematic review aims to address these gaps by clarifying inconsistencies in the reported associations between specific phospholipids and the aging process, investigating sex-specific differences in the plasma phospholipid profile associated with aging, and identifying metabolomic biomarkers of healthy versus pathological aging for potential clinical applications.

## Methods

 This systematic review examined the relationship between plasma phospholipids and SMs and aging in healthy men and women aged 60 years and older. The study was conducted in adherence to the Preferred Reporting Items for Systematic Reviews and Meta-Analyses (PRISMA) guidelines.^30^

###  Search strategy 

 A comprehensive search was conducted in MEDLINE (PubMed), Scopus, Embase, Web of Science, and Google Scholar for relevant articles up to September 2023, using appropriate keywords and MeSH terms. The detailed search strategy is provided in Supplementary file 1.

 Additionally, a manual search was performed through the reference and citation lists of the included studies and related review articles to identify relevant original research. All identified articles were imported into and managed using EndNote (version 8.1).

###  Inclusion and exclusion criteria

 Initially, two trained reviewers (MZ, MM) independently screened the articles based on their titles and abstracts. After excluding irrelevant studies, the remaining articles underwent a detailed full-text review to determine eligibility based on predefined criteria. Disagreements between reviewers were resolved through discussion with a third reviewer (MZ).

 The PECO framework for this study was defined as follows: participants included adults, the exposure was aging, comparisons involved control groups if applicable, and the outcome was changes in phospholipid levels. Cross-sectional and case-control studies were included if they investigated the relationship between aging and plasma SMs, PC, LPCs, and phosphatidylethanolamine (PE). The search was limited to English-language studies, and studies involving participants younger than 60 years of age, animal studies, conference abstracts, and review articles were excluded.

###  Quality assessment

 The quality of the included studies was evaluated independently by two reviewers using the Newcastle-Ottawa Scale (NOS).^31^ The NOS comprises three main sections: Selection, Comparability, and Outcome. Studies with NOS scores below five, between five and seven, and above seven were classified as having low, moderate, and high methodological quality, respectively.

###  Data extraction

 Two reviewers independently extracted data from all studies that met the inclusion criteria. The extracted information included the first author’s name, publication year, study location, target population, sex, participants’ age and BMI, number of participants, and serum lipid levels. The association between metabolites and aging was analyzed for both men and women. The third author verified the accuracy of the extracted data, and any disagreements between reviewers were resolved through consensus.

## Results

###  Study selection

**Figure 1:**
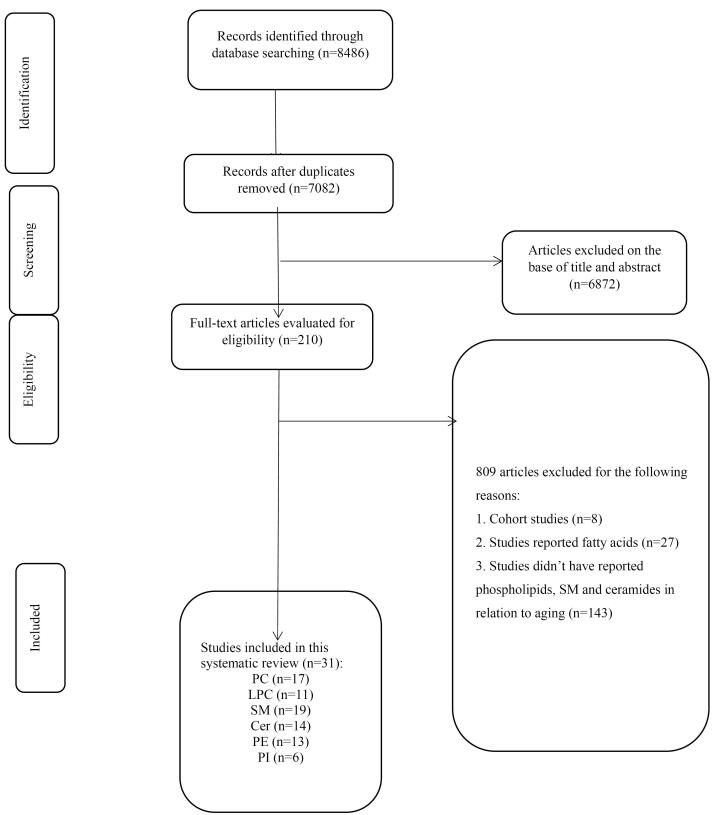
PRISMA flowchart of the study showing the study selection process

 As illustrated in the flowchart of the literature search and study selection process (Figure 1), the systematic search identified 8486 studies, of which 1404 were duplicates. The titles and abstracts of the remaining 7082 studies were screened, leading to the exclusion of 6872 studies. The full texts of the remaining 210 studies were then assessed for eligibility. Among these, 178 studies were excluded for the following reasons: eight were cohort studies, 27 focused on fatty acids rather than phospholipids, and 143 did not report data related to phospholipids in the context of aging. Ultimately, 31 studies met the specific inclusion criteria. Notably, the study by Zierer et al.^32^ summarized findings from two cohort studies, which were reported separately, and both datasets were included in the final analysis.

###  Study characteristics

 The characteristics of the eligible studies included in the analysis are presented in Table 1. Of the total studies, 26 were cross-sectional studies,^14,28,33-56^ while 5 were case-control studies.^57-61^ The present systematic review included 70,499 participants, with sample sizes ranging from 12 to 26,065. Twenty-eight studies included both sexes, while four studies focused exclusively on women and three exclusively on men. The mean age of participants across the included studies ranged from 20 to 90 years, and the mean BMI ranged from 20 to 28 kg/m^2^. The studies were published between 2013 and 2022. Most of the study populations were recruited from European countries, including four in the UK,^32,42,56,59^ three in Italy,^41,50,61^ and Netherlands,^42,62^ two in Germany,^35,42,53^ one in Spain,^51^ France,^52^ Finland,^14^ Switzerland,^36^ and eight in the US,^33,34,38,39,43,46,55,57^ five in Australia,^40,47,49,63,28^ two in Korea,^54,58^ and Japan,^45,48^ and one in Canada.^56^ The Target population of most included studies were healthy individuals and just five studies were carried out among Alzheimer’s patients^42,47,51,59^ and one among patients with diabetes and obesity.^40^ Three studies have reported changes in phospholipids in relation to telomere length.^32,33,42^ Four studies evaluated phospholipids in the brain tissue^47,49,51^ and the others evaluated in the blood of participants after an overnight fasting.

**Table 1:** Characteristics of included cohorts and reported changes in serum metabolites with aging

**First Author/year**	**Location**	**Study design**	**Population**	**Sample size**	**Age range**	**Main Phospholipid Focus**	**Key findings**	**Measurement Method**
Subedi, 2022^33^	USA	Cross-sectional	Patients with CVDs	1843	35-74	fatty acyls and sphingolipids, with specific mentions of palmitic acid and certain SMs	174 lipids were significantly linked with low telomere length, indicating a connection between altered lipid metabolism and biological ageing, independent of age, sex, and BMI.	LC-MS
Slade, 2021^34^	USA	Cross-sectional	Participants taking lipid-lowering medications	980	48.3	Associations examined across 413 lipid species Include classes like glycerolipids, GLPs, sphingolipids, sterol lipids, fatty acids, and acylcarnitines—specific focus on PCs, SMs	Significant age-associated increases in several lipid species, particularly PC and SM, demonstrate a direct relationship with ageing. Notable sex differences in lipid levels, with distinct profiles between males and females. Age and sex interactions were observed, suggesting differential lipidomic ageing profiles between genders	LC-MS
Carrard, 2021^36^	Switzerland	Cross-sectional	Clinically healthy individuals	150	Young female (25.1) Young male (25.1) Aged female (74.0) Aged male (73.9)	Emphasis on sphingolipids and GLPs, with notable changes in specific lipid species like ether-glycerophospholipids and lyso-glycerophosphocholine species in aged females	Significant increases in 138 and 107 lipid species for aged females and males respectively, including sphingolipids and glycerophospholipids. Certain lipid species, known to be cardiometabolic-ally favorable, were elevated in aged individuals	Liquid chromatography high-resolution mass spectrometry
Petrocelli, 2020^38^	USA	Cross sectional	Healthy population	35 participants (12 young adults, 23 older adults)	Young (23.4) Older (67.8)	Ceramides	Increasing in specific ceramide ratios such as C16:0/C24:0, C18:0/C24:0, and C24:1/C24:0 predominantly in older adults after periods of bed rest	HPLC coupled with tandem mass spectrometry
Cherrier, 2020^39^	USA	Cross sectional	Subjects drinking alcohol	183	Middle age: (35-59) Older adults: > 60	Phosphatidylethanolamine types	Consumption of higher amounts of alcohol in the middle-aged versus more days in the elderly. Increasing the concentration of both types of phosphatidyl ethanol in both middle-aged and old groups. Consumption of higher amounts of alcohol in the middle-aged versus more days in the elderly.	LC/MS
Beyene, 2020^40^	Australia	Cross-sectional	AusDiab, BHS	10339	25-95	PCs, PEs, PIs and SMs	Significant associations between age, sex, and BMI with various lipid species were identified. Specific lipid classes, such as ether-phospholipids and lysophospholipids, showed inverse associations with age in men only. Women displayed different lipidomic profiles, particularly after menopause, with changes in triacylglycerol and lysoalkyl-phosphatidylcholine species.	HPLC coupled with mass spectrometry
Wang, 2019^56^	Canada	Cross-sectional	Healthy subject	236	20 to 82	PCs, LPCs, SMs, and ceramides	The study revealed significant associations of ageing with specific phospholipids, notably sphingolipids and PCs, which varied less than 50% between sexes. Phospholipids associated with HDL particles increased, suggesting enhanced lipid transport functions that could improve cardiovascular health in older adults.	ESI-MS
Vozella, 2019^41^	Italy	Cross-sectional	post-menopausal women	100	47-78	The focus was on various ceramide molecules identified by their acyl chain lengths, such as Cer(d18:1/16:0) and Cer(d18:1/24:1)	The study found elevated levels of certain types of ceramides in post-menopausal women, which were associated with aging and hormonal changes Specific phospholipids measured include ceramides such as Cer(d18:1/16:0) and Cer(d18:1/24:1), which were significantly higher in older age groups.	HPLC tandem mass spectrometry
van der Spek, 2019^42^	Germany, Netherland, UK, Estonia	Cross-sectional	healthy adult populations	7853	Young (24–40 y) Older (75–90 y)	LPCs, PCs, and PEs	Phospholipids like LPC acyl C17:0, PC diacyl C32:1, and PC acyl-alkyl C38:4 were significantly associated with LTL. Higher levels of LPC a C17:0 and PC ae C38:4 were linked with longer LTL, indicating a protective role against biological ageing.	Mass spectrometry
Khayrullin, 2019^43^	USA	Cross- sectional	Healthy individuals	150	Young (24–40 y) Older (75–90 y)	SMs and PCs	during ageing certain SMs decreased, while some PCs increased.	LC-MS
Wong, 2019^28^	Australia	Cross- sectional	Healthy individuals	100	56–100 y	PCs, LPCs, SMs, PEs	Significant decline in various lipid species with age, with marked reductions in the oldest subjects suggesting a unique lipidomic profile associated with longevity. Notable differences in lipid profiles between males and females, with higher levels of certain lipid species in females	LC-MS
Wang, 2018^44^	UK	Cross-sectional and longitudinal study	Midlife women	3312	50	Circulating metabolic including a variety of lipids and phospholipids	Notable changes include increases in very low-density, intermediate-density, and low-density lipoprotein levels, and a decrease in lipoprotein particle size, indicating an increased cardiometabolic risk.	High-throughput NMR metabolomics
Kawanishi, 2018^45^	Japan	Cross-sectional	Adult subjects	20	Women: (Young 23.9) (Older 70.2) Men: (Young 23.9) (Older = 71.7)	Ester-linked PCs Ester-linked PEs Ether-linked PCs Ether-linked PEs	Ageing is associated with increased serum levels of multiple triacylglycerol species. Notable increases in total ester-linked PC and PE. Decreased serum levels of specific ether-linked PC and PE in elderly compared to the young.	LC-MS
Johnson, 2018^46^	USA	Cross-sectional	Healthy young and older adults	43	Young (23) Older (61)	Plasma, ceramides, and acyl carnitines	Specific phospholipids, notably ceramides, showed higher concentrations in older adults compared to younger adults. This included different ceramide chains such as C16:0, C18:0, C20:0, C22:0, C24:1, and C24:0, increases in certain ceramides were linked to declines in maximal oxygen consumption (V̇O2max), an important indicator of cardiovascular and overall physiological health	LC-MS
Díaz, 2018^51^	Spain	Cross-sectional	Alzheimer	25	Women: (Young 42.20) (Older 71.67) Men: (Young 42.86) (Older 73.29)	PIs, SMs, STs, and cerebrosides	Significant gender differences were observed in the aging process of lipid rafts, with these changes being more pronounced in women, especially postmenopausal. Lipid changes included alterations in the levels of phospholipids, sphingolipids, and cholesterol, which are essential for the functionality of lipid rafts	HPTLC
Couttas, 2018^47^	Australia	Cross-sectional	Alzheimer	80	78.2	ceramides, SMs, and ST and S1P	In males, ceramide, SM, and ST levels correlated positively with age. In females, an inverse correlation was observed between age and the S1P ratio, suggesting a decrease in neuroprotective sphingolipid levels with age.	LC-MS
Trabado, 2017^52^	French	Cross-sectional	Healthy individuals	800	37.6	PCs and SMs	Elderly subjects had higher levels of SMs and PCs compared to younger subjects, suggesting an increase in these phospholipids with age	low injection analysis (FIA) coupled to tandem mass spectrometry (MS/MS)
Rist, 2017^53^	Germany	Cross-sectional	Healthy men and women	301	18–80	PCs and SMs	Lack of definitive opinion on the relationship between the concentration of phospholipids and aging. Focusing on identifying metabolic patterns predictive of age and sex	Mass spectrometry coupled with gas chromatography or liquid chromatography, and NMR spectroscopy
Maekawa, 2017^48^	Japan	Cross-sectional	healthy Japanese adults	60	young males (25-35 years), elderly males (55-64 years), young females (25-35 years), and elderly females (55-65 years)	LPCs, PCs, ether-type PCs, ether-type PEs, and PIs	The study found age-associated differences in the levels of several phospholipids, with 111 (34%) and 115 (35%) metabolites showing significant age-related variations in males and females, respectively.	LC-MS
Zierer, 2016^32^	UK	Cross-sectional	women from the TwinsUK cohort	3511	53.6	1-stearoylglycerophosphoinositol and 1-palmitoylglycerophosphoinositol	Both 1-stearoylglycerophosphoinositol and 1-palmitoylglycerophosphoinositol were negatively associated with LTL (but non-significant), suggesting an increased phospholipase A2 (PLA2) activity and an altered membrane composition linked to biological ageing.	Gas chromatography/mass spectrometry and LC/MS
Norris, 2015 (B)^49^	Australia	Cross-sectional	Elderly individuals	36	58.9	PCs, PEs, and PS	Phospholipids containing docosahexaenoic acid (DHA) generally increased with age, whereas those containing arachidonic acid (AA) decreased. For example, mitochondrial PS 18:0_22:6 (which is high in DHA) increased significantly in abundance over the adult life span, while mitochondrial PE 18:0_20:4 (which contains AA) decreased.	Nano ESI-MS
Montoliu, 2014^50^	Italy	Cross-sectional	Healthy individuals	294	Elderly: (70.4) Centenarians: (100.7)	SMs, LPCs, PCs, and ether-linked PCs	Centenarians showed higher levels of certain SMs and ether-linked PCs, which may indicate enhanced antioxidant capacity and improved membrane lipid remodelling associated with healthy ageing. Specifically, increases in SM 36:2, SM 34:1, and PC-O species were noted, suggesting a protective role in maintaining cell integrity and possibly contributing to longevity	Shotgun MS/MS approach
Ishikawa, 2014^55^	USA	Cross-sectional	Healthy adults	60	Young: (M: 18.0–36.6) (F: 24.9–49.7) Elderly: (M: 19.5–34.9) (26.1–43.3)	LPCs, PCs ether-type PCs, PEs, ether-type PEs, and SMs	Age-associated differences in lipid metabolites were observed, particularly notable in females compared to males. In plasma and serum, many triacylglycerols were significantly higher in the elderly than in young females	UPLC-TOFMS
Auro, 2014^14^	Finland	Cross-sectional	Healthy population	26065	24–75	PCs and SMs	Significant gender-specific metabolic fingerprints were observed, with menopause associated with changes in lipid profiles, particularly the levels of PCs and SMs	NMR
Muilwijk, 2021^35^	Netherlands	Cross-sectional	Healthy population	700	46	Ceramides, lactosylceramides	Higher concentration of sphingolipids in young men compared to women and vice versa at older ages (56-70 years).	LC-tMS
Lee, 2014^54^	South Korea	Cross-sectional	Healthy Korean subjects	110	Young: 34.82 Elderly: 70.42	LPCs	There was a notable difference in the levels of specific phospholipids between the age groups. Notably, levels of LPCs were lower in the plasma of older individuals compared to the younger group, which could indicate membrane composition or metabolism alterations with age.	UPLC-QTOF-MS
Gonzalez-Covarrubias, 2013^62^	Netherlands	Case-control	Nonagenarian siblings of Caucasian descent	Offspring = 1526 Controls = 675	Offspring: 59.4 Control: 25.3	PCs, SMs, PEs	In women, 19 lipid species were significantly associated with familial longevity. Female offspring showed higher levels of ether PCs and SM species and lower levels of PEs (38:6).	Liquid chromatography coupled to mass spectrometry
D’Ascenzo, 2022^61^	Italy	Case-control	Parkinson’s Disease vs. healthy controls	78	73	PCs, SMs, ceramides, Lys phosphatidyls	Increasing concentration of PCs, SMs and ceramides in Parkinson's group compared to healthy people with increasing age and decreasing concentration of Lys phosphatidyls	LC-MS
Kim, 2017^59^	UK	Case-control	Alzheimer’s disease patients and controls	412	Alzheimer 77.35 Control 74.88	specific ceramides and PCs, including Cer16:0, Cer18:0, Cer24:1 for ceramides, and PC36:5, PC38:6 for	ceramides were associated with age, showing specific interactions with hippocampal atrophy particularly in younger participants (age < 75). PC36:5 was associated with AD status in the younger group, while PC38:6 was linked in the older group (age > 75).	UPLC-MS
Kim, 2019^58^	South Korea	Case-control	Healthy individuals	74	72	Ceramides C16:0, C18:0, C18:1, and C24:1	Age was positively correlated with plasma levels of C16:0, C18:0, and C24:1 ceramide. Patients with fragility hip fractures had significantly higher levels of C16:0, C18:0, C18:1, and C24:1 than those without fractures. These ceramides were positively associated with bone resorption markers. C18:0 and C24:1 ceramides were shown to directly increase osteoclastogenesis and bone resorption in vitro.	LC-tMS
Xyda, 2020^57^	USA	Case-control	Healthy population	24 participants (12 young adults, 12 older adults)	Younger: (27) Older: (76)	Impact of n3-PUFA on metabolomic profiles including lipoproteins and small metabolites	The relationship between phospholipid concentration and ageing showed that specific phospholipids associated with HDL particles increased, suggesting improved lipid transport functions in older adults, potentially aiding in better cardiovascular health outcomes.	Proton nuclear magnetic resonance (1H-NMR) and MS techniques

ST, sulfatide; S1P, sphingosine 1-phosphate; HPTLC, High-performance thin-layer chromatography; LC-MS, liquid chromatography-tandem mass spectrometry; NMR, nuclear magnetic resonance; AusDiab, Australian Diabetes, Obesity and Lifestyle Study; BHS, Busselton Health Study; CVD, cardiovascular disease; PC, phosphatidylcholine; SM, sphingomyelin; PE, phosphatidylethanolamines; PI, phosphatidylinositol; LPC, lysophosphatidylcholine; PS, phosphatidylserine; UPLC-QTOF-MS, Ultra-performance liquid chromatography coupled with quadrupole time-of-flight mass spectrometry; UPLC-TOFMS, Ultra-performance liquid chromatography-time of flight mass spectrometry; UPLC-MS, Mass spectrometry coupled with ultra-performance liquid chromatography

###  Quality assessment

 The NOS was used to assess the quality of the included studies, and the results are presented in Table 2. According to the NOS scoring system, most of the studies were classified as high-quality. The remaining 15 studies were rated as having moderate quality. Additionally, the statistical analyses, assessed as part of the outcome component in the NOS, were thoroughly described in all studies.

**Table 2:** Quality assessment of included studies using Newcastle-Ottawa Scale

**First author, Year**	**Selection**				**Comparability**	**Exposure**		**Total score**
	**Item 1**	**Item 2**	**Item 3**	**Item 4**	**Item 1**	**Item 1**	**Item 2**	
Subedi, 2022^33^	*	*	*	**	**	**	*	10
D’Ascenzo, 2022^61^	*	**-**	**-**	**	**-**	*	*	5
Muilwijk, 2021^35^	*	*	*	**	**	**	*	10
Slade, 2021^33^	*	*	*	**	**	**	*	10
Carrard, 2021^36^	*	**-**	*	**	**	*	*	8
Xyda, 2020^57^	*	**-**	**-**	**	**	**	*	8
Petrocelli, 2020^38^	*	**-**	**-**	**	**-**	**	*	6
Cherrier, 202039	*	**-**	**-**	**	**-**	*	*	5
Beyene, 2020^41^	*	*	*	**	**	**	*	10
Wang, 2019^56^	*	*	*	**	*	**	*	9
Vozella, 2019^41^	*	**-**	**-**	**	**	*	*	7
van der Spek, 2019^42^	*	*	*	**	**	**	*	10
Kim, 2019^58^	*	**-**	**-**	**	**	**	*	8
Khayrullin, 2019^43^	-	-	*	**	*	-	*	5
Wong, 2019^28^	*	*	*	**	*	**	*	9
Wang, 2018^44^	*	*	*	**	**	**	*	10
Kawanishi, 2018^45^	*	**-**	**-**	**	**-**	**	*	6
Johnson, 2018^46^	*	-	*	*	*	*	*	6
Díaz, 2018^51^	*	*	*	*	*	-	*	6
Couttas, 2018^47^	*	*	*	*	*	*	*	7
Trabado, 2017^52^	*	*	*	**	*	-	*	7
Rist, 2017^53^	*	*	*	**	*	*	*	8
Maekawa, 2017^48^	*	*	*	**	**	*	*	9
Kim, 2017^59^	*	*	*	**	**	**	*	10
Zierer, 2016^32^	*	*	*	**	**	**	*	10
Norris, 2015^49^	-	*	*	*	*	*	*	6
Montoliu, 2014^50^	*	*	*	*	*	*	*	7
Ishikawa, 2014^55^	*	*	*	**	**	*	*	9
Auro, 2014^14^	*	*	*	**	*	*	*	8
Lee, 2014^54^	*	-	*	**	-	*	*	6
Gonzalez-Covarrubias, 2013^62^	*	*	*	**	*	*	*	8

NOS comprises from three domains: Selection, Comparability, Outcome. Selection domain includes three items: Representativeness of the sample, Sample size, Non-respondents and Ascertainment of the exposure (risk factor). Comparability domain includes: The subjects in different outcome groups are comparable, based on the study design or analysis. Confounding factors are controlled item. Outcome domain includes two items: Assessment of the outcome and Statistical test. *Means that the study obtained one score from each item. **Means that the study obtained two score from each item.

###  Changes in phospholipids during aging

 The results of changes in phospholipids are presented in the Table 1 and the direction of aging effects on these phospholipids has been illustrated.

####  Phosphatidylcholines 

 A category of lipids impacted by aging was PC. PCs were assessed in seventeen studies, all of which included both male and female participants combined.^14,28,33,34,40,42,45,48-52,56,57,59,61,62^ Three studies have indicated that PCs are positively associated with aging process in both genders (*P* < 0.05).^34,48,49^ In contrast, Wong et al reported significant inverse relationship between PCs and aging trend among both genders.^28^ While, Kawanishi et al^45^ have shown that (PC 32:0, PC 34:2, PC 34:3, PC 36:5, PC 38:2, PC 38:5, PC 38:6, PC 40:5, PC 40:6, PC 40:7) were positively associated with aging among men (*P* < 0.05). However, women showed different results for different PCs and PC 32:1, PC 36:5, PC 38:2, PC 38:5, PC 40:5, PC 40:6, PC 40:7 were inversely associated with aging among healthy elderly women (*P* < 0.05).^45^ Similarly, Beyene et al represented results in terms of PCs in association with aging among Australian obese and diabetes patients in a sex-stratified model. PC levels showed a significant positive and negative relationship with age among men and women respectively.^40^ Whereas, Montoliu et al reported different findings for different PCs in association with the aging process. In such a way that PCs (14:0-18:1), (16:0-18:3), (18:0-22:5) tended to show an upward trend according to aging (*P* < 0.05) in contrast to PCs (16:0-18:1) and (16:0-18:2) (*P* < 0.05).^50^ In contrast, four other studies that have evaluated the behavior of PCs in relation to aging, found no significant relationship between PCs and aging trend.^14,51,52,61^

 The studies of Kim et al^57^ and Xyda et al^57^ had a case-control design. Decreased levels of PC were not significantly associated with the aging process among AD patients compared to the control group in the study conducted by Kim et al.^59^ Xyda et al examined the behavior of PCs in the context of aging and found no significant association between PCs and aging trends similar to others.^57^

 In addition, in some studies, PC metabolites were assessed too. The ether form of PCs [PC (O)] was negatively associated with aging in both genders in two studies^40,62^ and just among women in one study.^45^ Similarly, alkenyl phospholipids [PC (P)] were negatively associated with the aging process in both genders in Beyene and colleagues’ study (*P* < 0.05).^40^ Furthermore, diacyl PC and acyl-alkyl PC including aaC34:1, aaC32:0, aaC32:2, aaC34:2, aaC36:2, aeC44:4 and aeC42:4 were elevated in Parkinson’s patients vs control group in Ascenzo’s study.^61^ Ashley et al have investigated the correlation between PCs and leucocyte telomere length among AD patients and found that the PC aa C32:1 and PC ae C38:4 significantly decreased and increased, respectively, during aging in AD patients.^42^ However, PCs were associated with telomere length in Subedi and colleagues’ study.^33^

####  Lysophosphatidylcholines

 LPC levels were reported in eleven studies with different results.^34,40,42,48,49,51,53-55,57,61^ Lee et al^54^ reported a significant and positive association between LPCs and the aging process among both genders. Also, in Maekawa’s study, the metabolite of LPC followed a similar trend, and LPC metabolite was significantly increased in old women compared to younger ones.^48^ Also, Ishikawa et al^55^ reported contradictory results in different LPCs classes. They have reported significantly increased levels of LysoPC 16:1, LysoPC 17:0, LysoPC 18:0 and LysoPC 22:6 in men and significantly decreased levels of LysoPC 16:1, LysoPC 16:0, LysoPC 18:0, and LysoPC 22:6 in elderly women compared to younger women. Moreover, Ashley et al reported a positive association of LPCs with telomere length.^42^ While other studies have reported a significant decreasing trend for LPCs in both genders in plasma^40^ and tissue levels.^49^ While, four other studies which have evaluated the LPCs characteristics in relation to aging, have found no significant relationships in both plasma^34,51,53,57^ and tissue levels. It should be noted that the study by Xyda et al was a case-control study.^57^ In addition, PD patients showed lower levels of LPCs in comparison to the control group irrespective of the aging effect too.^61^

####  Sphingomyelin and ceramides 

 The SMs levels were evaluated in nineteen studies.^14,28,33,34,36,40,42,45-48,50,51,53,54,56,61,62,52^ Three studies have reported that SM levels are positively correlated with the aging trend in both genders (*P* < 0.05).^34,40,62^ This relationship was also true for its metabolites. SM’s metabolites were increased in old population compared to younger in both genders (*P* < 0.05).^48^ Similarly, Couttas et al have reported this positive association of SMs in men specifically.^47^ Also, levels of SM (42:4), SM (42:3), SM (42:2), SM (41:2), SM (38:2), SM (36:2), SM (36:1), SM (34:1) and SM (33:1) except SM (50:1) were increased in centenarians compared to elderlies (*P* < 0.05).^50^ Whereas, two other studies reported contradictory results indicating an inverse relationship of SM with the aging trend (*P* < 0.05).^54,28^ Contrary, seven other studies that have evaluated the behaviors of SMs in relation to aging, found no significant association in this regard.^36,42,45,46,51-53^ Regardless of aging effects, hydro-SMs were elevated in PD patients vs. the control group.^61^ In addition, SM (d42:3) and SM (d18:0/24:1) were in positive and negative association with telomere length, respectively.^33^

 Furthermore, fourteen studies reported on ceramides in relation to aging.^28,33-36,38,40,41,43,46-48,58,59,33^ Eight studies have reported elevated levels of ceramides in aged individuals compared to younger subjects (*P* < 0.05).^38,40,41,43,46-48,58^ In contrast, Wong et al demonstrated decreased levels of ceramides in elderly vs young people (*P* < 0.05).^28^ It is worth noting that Khayrullin et al evaluated ceramide levels in serum exosome of women and (C16:0), (C18:0 Cer), (C24:1 Cer), (C24:1 Cer) were positively correlated with aging (*P* < 0.05).^43^ Also, total ceramide among men was positively correlated with aging (*P* < 0.05).^47^ However, Alzheimer’s patients and healthy subjects showed elevated levels of ceramides in Kim et al study.^59^

####  Phosphatidylethanolamines 

 Thirteen published studies conducted among healthy individuals, have studied PE level.^28,34,36,39,40,45,46,48-51,62^ Five studies demonstrated a significant positive association between PE levels and aging.^34,40,48,50,62^ In contrast, PE levels were inversely associated with aging in two other studies (*P* < 0.05).^39,28^ While, Johnson and Diaz et al. found no changes in term of PEs with respect to the aging process.^46,51^ Kawanishi et al demonstrated increased levels of PE 36:1, PE 38:6, PE 40:6 in elderly women and PE 36:1, PE 36:2, PE 36:3 in elderly men vs young.^45^ Similarly, PEs were in a positive relationship with aging except PE (18:1) in Norris and colleagues’ study.^49^ In addition, alkyl PE (PE-O) the ether form of PEs, showed a positive association in the tissue of Australian elderly people^49^ and a negative association with aging in Beyene et al and Norris and colleagues’ studies.^40,45^ Also, LPE levels illustrated an upward trend according to aging in two studies.^34,40^ Moreover, lysoalkylglycero PE was seen in a positive association with aging.^36^

####  Phosphatidylinositol 

 Another class of lipids that altered due to aging was the PIs. Three studies have reported significantly increasing levels of PI in aged people compared to young ones.^34,40,48,50^ While it was decreased among the healthy UK population in Zierer’s study.^32^

## Discussion

 This study addresses a critical knowledge gap regarding the inconsistent findings on the relationship between plasma phospholipids and aging, particularly in relation to variations in phospholipid classes across sex, BMI categories, and tissue types. While previous research has investigated this association, many studies lack detailed stratification by sex or health conditions, limiting the broader applicability of their conclusions. This review fills that gap by systematically examining cross-sectional and case-control studies to shed light on sex-specific differences in phospholipid profiles and to identify key biomarkers linked to both healthy and pathological aging.

 Moreover, this study deepens the understanding of lipid-related mechanisms involved in the aging process and highlights the potential for metabolomic biomarkers to play a role in the prevention and management of age-related diseases. By integrating findings from diverse populations, this review provides a more comprehensive perspective and contributes valuable new insights to the existing literature.

 Overall, the findings from the included studies suggest that the relationship between various phospholipids and aging depends on tissue type, sex, BMI, fatty acid saturation, and the specific phospholipid class. However, a systematic review of cohort studies by Mohammadzadeh Honarvar et al concluded that sex is a more significant factor in determining phospholipid levels during aging.^64^ However, the findings of our study indicate that there is no consistent pattern for determining the relationship between phospholipids and sex, as this relationship can be influenced by the aforementioned factors. PCs are the most abundant type of phospholipid in cells, accounting for approximately 50% of the total phospholipid content. They are primarily found in the endoplasmic reticulum and, to a lesser extent, in the cell membrane.^65^ Therefore, maintaining adequate levels of PCs is essential for normal cellular function. A reduction in total PC levels is both a biomarker and a contributing factor in the aging process. Kim et al demonstrated that in vivo supplementation with PCs extended lifespan by influencing the nuclear localization of DAF-16, a transcription factor involved in the stress response.^66^ Overall, observational studies have indicated a significant relationship between PC levels and aging. Maekawa et al identified PC metabolites as the primary age-dependent phospholipids.^48^ However, it seems that the inconsistency of the results was related to studied PC and examined tissue. While total PCs levels decreased with aging, brain levels increased. Three studies evaluated PCs in the brain tissue, of which two studies showed a significant increase of PCs in brain tissue by aging^49^ and one failed to report a significant change.^51^ Among the different types of PC, the unsaturated forms may have distinct effects on the aging process compared to the saturated forms. Some in vivo studies have suggested that a reduction in certain unsaturated PCs may contribute to lifespan extension.^67,68^ However, the findings of our study suggest that an increase in certain unsaturated PCs may be associated with longevity. Additionally, ether-linked phospholipids have demonstrated antioxidant properties and may help protect myelin from oxidative damage.^69^ As reported by Beyene et al and Gonzalez-Covarrubias, ether-linked phospholipids were inversely associated with aging.^40,62^ However, diacyl PCs and acyl-alkyl PCs were elevated in Parkinson’s patients in Ascenzo and colleagues’ study.^61^

 LPCs play a critical role in the cardiolipin biosynthesis pathway, serving as an essential component of mitochondrial membranes. Consequently, LPCs contribute to the regulation of mitochondrial oxidative capacity.^70^ Mitochondrial dysfunction is involved in aging process. As a result, low level of LPCs can predict aging phenotypes such as myocardial infarction^71^ and cognitive impairment.^72^ Similar to this hypothesis, Beyene et al have shown that there was a decreasing trend for LPCs in both gender in plasma by aging.^40^ Moreover, a positive association was found between leukocyte telomere length and LPCs level in AD patients.^42^ In contrast, Lee et al suggested that higher plasma levels of LPCs can be a possible cause of neuroinflammation and atherosclerosis by aging.^54^ According to the findings of a review study, due to the extensive interaction of LPCs with immune cells, LPCs cannot be considered simply as pro-inflammatory or anti-inflammatory agents.^73^ These conflicting results can be related to the length of the acyl chain, the degree of saturation, as well as the age and health status of the subjects. As a result, similar to PCs, the relationship of LPCs with aging is complex and does not follow a simple fashion.

 Ceramide and SM can be interconverted through the actions of SM synthase and sphingomyelinase, respectively.^74^ Sphingomyelinase can be activated by various stimuli such as oxidative stress and various cytokines.^75^ Increased oxidative stress, as a natural consequence of the aging process, has been linked to ceramide accumulation. Studies have shown that ceramide levels rise during the early stages of AD but exhibit a global decline in the later stages of the condition.^76^ However, similar to PCs and LPCs, ceramides with varying acyl chain lengths exhibit different responses to the aging process. Cutler et al reported that very long-chain ceramides tend to accumulate with age.^77^ Moreover, long-chain ceramide accumulation leads to mitochondrial dysfunction and, subsequently oxidative stress and cell death.^78,79^ Various observational studies have confirmed the above findings on ceramides.^38,40,42,43,46,47,58,59^ However, Wong et al. revealed a negative association between ceramides and aging. An evaluation of their findings showed that subjects aged 95 and above experienced a significant reduction in ceramides^28^; while other studies mainly included middle-aged adults. This contradictory finding may be attributed to the age groups of the studied subjects. Overall, the results indicate that SM levels generally increase with aging; however, some conflicting findings have been reported. The reduction in SM levels observed in Wong and colleagues’ study may be explained by the same age-related factor mentioned earlier.^28^ Regarding the study by Lee et al,^54^ the low level of SM could be related to the low BMI of the participants. Also, assessing other conflicting results showed that the response of SM to the aging process can be different between women and men. However, more studies are needed to clarify this issue.

 Reduction of PE, as the second abundant phospholipid in organisms, has been suggested a general feature of aging by *in-vivo* studies.^80-82^ Park et al reported that supplementation with PE led to anti-oxidant and anti-aging effects through the reduction of insulin/IGF-1-like signaling in C. elegans.^83^ However, similar to PCs, Beyene et al. and Kawanishi et al. reported that only ether-linked PEs were negatively associated with aging.^40,45^ Moreover, Norris et al. indicated that PEs containing docosahexaenoic acid 22:6 (DHA) were elevated in brain tissue by aging.^49^ Considering the involvement of DHA in the growth and function of neurons, this process can be considered as an adaptive and protective mechanism for the aging process in the brain. Another effective factor is age. Montoliu et al. found a positive association between PEs and aging in centenarians compared with elderly subjects.^50^ The difference between centenarians and younger elders is their ability to balance the pro-inflammatory and anti-inflammatory state.^84^ With the mentioned findings, it can be found that one of the underlying mechanisms in this issue is the increase in PEs and reduction in ceramides in centenarians.

 PI plays a critical role in cell signaling. Through this effect on cell signaling, it has been suggested that PIs possess anti-inflammatory activities by inhibition of protein kinase C (PKC)- mitogen-activated protein kinase (MAPK) pathways.^85^ This can justify higher level of PIs in centenarians compared to elderly subjects and their ability to maintain the balance between anti- and pro-inflammatory eicosanoids.^50^ In contrast, a positive association was found between PIs and aging in a study by Slade et al. This contradictory result can be related to the use of lipid-lowering drugs in their studied subjects.^34^ Moreover, BMI of participants can explain other contradictory results.^36^ It has been suggested that PI derivate, PI(4,5)P2, could enhance lifespan by affecting *daf-18* (as a tumor suppressor gene); on the other hand, PI(3,4,5)P3 through affecting insulin and insulin-like growth factor signaling pathway had a shortening effect on lifespan.^86^ Therefore, the function of enzymes involved in the metabolism of PIs can affect the aging process and be considered as future therapeutic targets in aging studies. As reported by Matuoka et al, phosphatidylinositol 3-kinase (PI3K) activity can promote aging phenotypes.^87^ As a result, the activity of enzymes involved in the metabolism of PIs seems to be more important than the level of PI metabolites.

## Strengths and limitations

 The strength of this manuscript lies in its comprehensive systematic review of 32 studies, conducted following PRISMA guidelines. It provides an in-depth examination of the relationship between phospholipids and aging, highlighting key trends while accounting for gender differences and methodological variability. However, the study has several limitations, including a lack of research on the effects of lipid-lowering medications, substantial variability in measurement techniques, population heterogeneity, and an inability to perform a meta-analysis due to high levels of heterogeneity.

 Additionally, factors such as circadian rhythms, fasting status, differences in laboratory methods and assay kits, and variations in BMI may have influenced the results. Further research is needed on centenarians and the mechanisms underlying the impact of phospholipids on the aging process. Moreover, the absence of essential data in many of the included studies prevented the performance of a full analysis and the presentation of a meta-analysis.

###  Clinical recommendations for future studies

 Phospholipid monitoring, particularly for PCs and LPCs, could serve as biomarkers for aging-related diseases like cardiovascular and cognitive decline, and should be considered in elderly patients. Given gender and age-based lipid variations, personalized treatments may be needed to manage aging-related conditions more effectively. Further research is required to clarify how specific phospholipids influence aging and related diseases, especially in centenarians and individuals on lipid-lowering medications. Investigating the impact of lipid-lowering drugs on phospholipid levels in aging populations could help tailor therapies for older adults. Future studies should also account for circadian rhythms and fasting times to standardize lipid measurements. Expanding research to include diverse populations, such as centenarians and individuals with varying BMIs, will improve our understanding of how phospholipid profiles change across different demographics.

## Conclusion

 Phospholipids have crucial roles in the aging process, aging-related diseases, and lifespan regulation. Decreased total levels of PCs, LPCs, and Pes and accumulation of ceramides are the features of aging. However, the examined tissue, gender, BMI, saturation of FA, and type of phospholipid can affect this pattern. Activity of enzymes involved in the metabolism of PIs especially PI3K seems to be more important than the level of PI metabolites.

## Competing Interests

 None.

## Data Availability Statement

 The data will be made available upon request.

## Ethical Approval

 Not applicable.

## Supplementary Files

Supplementary file 1. The search pattern used for search in PubMed database.

## References

[1] Deelen J, Beekman M, Uh HW, Helmer Q, Kuningas M, Christiansen L (2011). Genome-wide association study identifies a single major locus contributing to survival into old age; the APOE locus revisited. Aging Cell.

[2] Karasik D, Demissie S, Cupples LA, Kiel DP (2005). Disentangling the genetic determinants of human aging: biological age as an alternative to the use of survival measures. J Gerontol A Biol Sci Med Sci.

[3] Kerber RA, O’Brien E, Cawthon RM (2009). Gene expression profiles associated with aging and mortality in humans. Aging Cell.

[4] Piper MD, Bartke A (2008). Diet and aging. Cell Metab.

[5] Weckwerth W (2003). Metabolomics in systems biology. Annu Rev Plant Biol.

[6] Wenk MR (2005). The emerging field of lipidomics. Nat Rev Drug Discov.

[7] Ference BA, Graham I, Tokgozoglu L, Catapano AL (2018). Impact of lipids on cardiovascular health: JACC health promotion series. J Am Coll Cardiol.

[8] Roche HM (2005). Fatty acids and the metabolic syndrome. Proc Nutr Soc.

[9] Shen J, He J, Wang F (2016). Association of lipids with age-related macular degeneration. Discov Med.

[10] Wong MW, Braidy N, Poljak A, Pickford R, Thambisetty M, Sachdev PS (2017). Dysregulation of lipids in Alzheimer’s disease and their role as potential biomarkers. Alzheimers Dement.

[11] Holmes MV, Millwood IY, Kartsonaki C, Hill MR, Bennett DA, Boxall R (2018). Lipids, lipoproteins, and metabolites and risk of myocardial infarction and stroke. J Am Coll Cardiol.

[12] Yu Z, Zhai G, Singmann P, He Y, Xu T, Prehn C (2012). Human serum metabolic profiles are age dependent. Aging Cell.

[13] Darst BF, Koscik RL, Hogan KJ, Johnson SC, Engelman CD (2019). Longitudinal plasma metabolomics of aging and sex. Aging (Albany NY).

[14] Auro K, Joensuu A, Fischer K, Kettunen J, Salo P, Mattsson H (2014). A metabolic view on menopause and ageing. Nat Commun.

[15] Dorninger F, Moser AB, Kou J, Wiesinger C, Forss-Petter S, Gleiss A (2018). Alterations in the plasma levels of specific choline phospholipids in Alzheimer’s disease mimic accelerated aging. J Alzheimers Dis.

[16] Mielke MM, Bandaru VV, Han D, An Y, Resnick SM, Ferrucci L (2015). Factors affecting longitudinal trajectories of plasma sphingomyelins: the Baltimore Longitudinal Study of Aging. Aging Cell.

[17] Bergman BC, Brozinick JT, Strauss A, Bacon S, Kerege A, Bui HH (2015). Serum sphingolipids: relationships to insulin sensitivity and changes with exercise in humans. Am J Physiol Endocrinol Metab.

[18] Haus JM, Kashyap SR, Kasumov T, Zhang R, Kelly KR, Defronzo RA (2009). Plasma ceramides are elevated in obese subjects with type 2 diabetes and correlate with the severity of insulin resistance. Diabetes.

[19] Lemaitre RN, Yu C, Hoofnagle A, Hari N, Jensen PN, Fretts AM (2018). Circulating sphingolipids, insulin, HOMA-IR, and HOMA-B: the strong heart family study. Diabetes.

[20] Laaksonen R, Ekroos K, Sysi-Aho M, Hilvo M, Vihervaara T, Kauhanen D (2016). Plasma ceramides predict cardiovascular death in patients with stable coronary artery disease and acute coronary syndromes beyond LDL-cholesterol. Eur Heart J.

[21] Lemaitre RN, Jensen PN, Hoofnagle A, McKnight B, Fretts AM, King IB (2019). Plasma ceramides and sphingomyelins in relation to heart failure risk. Circ Heart Fail.

[22] Muilwijk M, Goorden SMI, Celis-Morales C, Hof MH, Ghauharali-van der Vlugt K, Beers-Stet FS (2020). Contributions of amino acid, acylcarnitine and sphingolipid profiles to type 2 diabetes risk among South-Asian Surinamese and Dutch adults. BMJ Open Diabetes Res Care.

[23] Peterson LR, Xanthakis V, Duncan MS, Gross S, Friedrich N, Völzke H (2018). Ceramide remodeling and risk of cardiovascular events and mortality. J Am Heart Assoc.

[24] Park JW, Park WJ, Futerman AH (2014). Ceramide synthases as potential targets for therapeutic intervention in human diseases. Biochim Biophys Acta.

[25] Chatterjee S, Bedja D, Mishra S, Amuzie C, Avolio A, Kass DA (2014). Inhibition of glycosphingolipid synthesis ameliorates atherosclerosis and arterial stiffness in apolipoprotein E-/- mice and rabbits fed a high-fat and -cholesterol diet. Circulation.

[26] Weir JM, Wong G, Barlow CK, Greeve MA, Kowalczyk A, Almasy L (2013). Plasma lipid profiling in a large population-based cohort. J Lipid Res.

[27] Mielke MM, Bandaru VV, Han D, An Y, Resnick SM, Ferrucci L (2015). Demographic and clinical variables affecting mid- to late-life trajectories of plasma ceramide and dihydroceramide species. Aging Cell.

[28] Wong MW, Braidy N, Pickford R, Vafaee F, Crawford J, Muenchhoff J (2019). Plasma lipidome variation during the second half of the human lifespan is associated with age and sex but minimally with BMI. PLoS One.

[29] Mihalik SJ, Goodpaster BH, Kelley DE, Chace DH, Vockley J, Toledo FG (2010). Increased levels of plasma acylcarnitines in obesity and type 2 diabetes and identification of a marker of glucolipotoxicity. Obesity (Silver Spring).

[30] Page MJ, McKenzie JE, Bossuyt PM, Boutron I, Hoffmann TC, Mulrow CD (2021). The PRISMA 2020 statement: an updated guideline for reporting systematic reviews. BMJ.

[31] Stang A (2010). Critical evaluation of the Newcastle-Ottawa scale for the assessment of the quality of nonrandomized studies in meta-analyses. Eur J Epidemiol.

[32] Zierer J, Kastenmüller G, Suhre K, Gieger C, Codd V, Tsai PC (2016). Metabolomics profiling reveals novel markers for leukocyte telomere length. Aging (Albany NY).

[33] Subedi P, Palma-Gudiel H, Fiehn O, Best LG, Lee ET, Howard BV (2023). Lipidomics profiling of biological aging in American Indians: the Strong Heart Family Study. Geroscience.

[34] Slade E, Irvin MR, Xie K, Arnett DK, Claas SA, Kind T (2021). Age and sex are associated with the plasma lipidome: findings from the GOLDN study. Lipids Health Dis.

[35] Muilwijk M, Callender N, Goorden S, Vaz FM, van Valkengoed IG (2021). Sex differences in the association of sphingolipids with age in Dutch and South-Asian Surinamese living in Amsterdam, the Netherlands. Biol Sex Differ.

[36] Carrard J, Gallart-Ayala H, Infanger D, Teav T, Wagner J, Knaier R (2021). Metabolic view on human healthspan: a lipidome-wide association study. Metabolites.

[37] Zhang X, Wang T, Song J, Deng J, Sun Z (2020). Study on follicular fluid metabolomics components at different ages based on lipid metabolism. Reprod Biol Endocrinol.

[38] Petrocelli JJ, McKenzie AI, Mahmassani ZS, Reidy PT, Stoddard GJ, Poss AM (2020). Ceramide biomarkers predictive of cardiovascular disease risk increase in healthy older adults after bed rest. J Gerontol A Biol Sci Med Sci.

[39] Cherrier MM, Shireman LM, Wicklander K, Yeung W, Kooner P, Saxon AJ (2020). Relationship of phosphatidylethanol biomarker to self-reported alcohol drinking patterns in older and middle-age adults. Alcohol Clin Exp Res.

[40] Beyene HB, Olshansky G, Smith AA, Giles C, Huynh K, Cinel M (2020). High-coverage plasma lipidomics reveals novel sex-specific lipidomic fingerprints of age and BMI: evidence from two large population cohort studies. PLoS Biol.

[41] Vozella V, Basit A, Piras F, Realini N, Armirotti A, Bossù P (2019). Elevated plasma ceramide levels in post-menopausal women: a cross-sectional study. Aging (Albany NY).

[42] van der Spek A, Broer L, Draisma HH, Pool R, Albrecht E, Beekman M (2019). Metabolomics reveals a link between homocysteine and lipid metabolism and leukocyte telomere length: the ENGAGE consortium. Sci Rep.

[43] Khayrullin A, Krishnan P, Martinez-Nater L, Mendhe B, Fulzele S, Liu Y (2019). Very long-chain C24:1 ceramide is increased in serum extracellular vesicles with aging and can induce senescence in bone-derived mesenchymal stem cells. Cells.

[44] Wang Q, Ferreira DLS, Nelson SM, Sattar N, Ala-Korpela M, Lawlor DA (2018). Metabolic characterization of menopause: cross-sectional and longitudinal evidence. BMC Med.

[45] Kawanishi N, Kato Y, Yokozeki K, Sawada S, Sakurai R, Fujiwara Y (2018). Effects of aging on serum levels of lipid molecular species as determined by lipidomics analysis in Japanese men and women. Lipids Health Dis.

[46] Johnson LC, Martens CR, Santos-Parker JR, Bassett CJ, Strahler TR, Cruickshank-Quinn C (2018). Amino acid and lipid associated plasma metabolomic patterns are related to healthspan indicators with ageing. Clin Sci (Lond).

[47] Couttas TA, Kain N, Tran C, Chatterton Z, Kwok JB, Don AS (2018). Age-dependent changes to sphingolipid balance in the human hippocampus are gender-specific and may sensitize to neurodegeneration. J Alzheimers Dis.

[48] Maekawa K, Okemoto K, Ishikawa M, Tanaka R, Kumagai Y, Saito Y (2017). Plasma lipidomics of healthy Japanese adults reveals gender- and age-related differences. J Pharm Sci.

[49] Norris SE, Friedrich MG, Mitchell TW, Truscott RJ, Else PL (2015). Human prefrontal cortex phospholipids containing docosahexaenoic acid increase during normal adult aging, whereas those containing arachidonic acid decrease. Neurobiol Aging.

[50] Montoliu I, Scherer M, Beguelin F, DaSilva L, Mari D, Salvioli S (2014). Serum profiling of healthy aging identifies phospho- and sphingolipid species as markers of human longevity. Aging (Albany NY).

[51] Díaz M, Fabelo N, Ferrer I, Marín R (2018). “Lipid raft aging” in the human frontal cortex during nonpathological aging: gender influences and potential implications in Alzheimer’s disease. Neurobiol Aging.

[52] Trabado S, Al-Salameh A, Croixmarie V, Masson P, Corruble E, Fève B (2017). The human plasma-metabolome: reference values in 800 French healthy volunteers; impact of cholesterol, gender and age. PLoS One.

[53] Rist MJ, Roth A, Frommherz L, Weinert CH, Krüger R, Merz B (2017). Metabolite patterns predicting sex and age in participants of the Karlsruhe Metabolomics and Nutrition (KarMeN) study. PLoS One.

[54] Lee SH, Park S, Kim HS, Jung BH (2014). Metabolomic approaches to the normal aging process. Metabolomics.

[55] Ishikawa M, Maekawa K, Saito K, Senoo Y, Urata M, Murayama M (2014). Plasma and serum lipidomics of healthy white adults shows characteristic profiles by subjects’ gender and age. PLoS One.

[56] Wang Y, Wang G, Jing Rn, Hu T, Likhodii S, Sun G (2020). Metabolomics analysis of human plasma metabolites reveals the age- and sex-specific associations. J Liq Chromatogr Relat Technol.

[57] Xyda SE, Vuckovic I, Petterson XM, Dasari S, Lalia AZ, Parvizi M (2020). Distinct influence of omega-3 fatty acids on the plasma metabolome of healthy older adults. J Gerontol A Biol Sci Med Sci.

[58] Kim BJ, Lee JY, Park SJ, Lee SH, Kim SJ, Yoo HJ (2019). Elevated ceramides 18:0 and 24:1 with aging are associated with hip fracture risk through increased bone resorption. Aging (Albany NY).

[59] Kim M, Nevado-Holgado A, Whiley L, Snowden SG, Soininen H, Kloszewska I (2017). Association between plasma ceramides and phosphatidylcholines and hippocampal brain volume in late onset Alzheimer’s disease. J Alzheimers Dis.

[60] Gonzalez-Covarrubias V, Beekman M, Uh HW, Dane A, Troost J, Paliukhovich I (2013). Lipidomics of familial longevity. Aging Cell.

[61] D’Ascenzo N, Antonecchia E, Angiolillo A, Bender V, Camerlenghi M, Xie Q (2022). Metabolomics of blood reveals age-dependent pathways in Parkinson’s Disease. Cell Biosci.

[62] Gonzalez-Covarrubias V (2013). Lipidomics in longevity and healthy aging. Biogerontology.

[63] Hancock SE, Friedrich MG, Mitchell TW, Truscott RJ, Else PL (2017). The phospholipid composition of the human entorhinal cortex remains relatively stable over 80 years of adult aging. Geroscience.

[64] Mohammadzadeh Honarvar N, Zarezadeh M, Molsberry SA, Ascherio A (2021). Changes in plasma phospholipids and sphingomyelins with aging in men and women: A comprehensive systematic review of longitudinal cohort studies. Ageing Res Rev.

[65] van Meer G, Voelker DR, Feigenson GW (2008). Membrane lipids: where they are and how they behave. Nat Rev Mol Cell Biol.

[66] Kim SH, Kim BK, Park S, Park SK (2019). Phosphatidylcholine extends lifespan via DAF-16 and reduces amyloid-beta-induced toxicity in Caenorhabditis elegans. Oxid Med Cell Longev.

[67] Wan QL, Yang ZL, Zhou XG, Ding AJ, Pu YZ, Luo HR (2019). The effects of age and reproduction on the lipidome of Caenorhabditis elegans. Oxid Med Cell Longev.

[68] He B, Xu J, Pang S, Tang H (2021). Phosphatidylcholine mediates the crosstalk between LET-607 and DAF-16 stress response pathways. PLoS Genet.

[69] Luoma AM, Kuo F, Cakici O, Crowther MN, Denninger AR, Avila RL (2015). Plasmalogen phospholipids protect internodal myelin from oxidative damage. Free Radic Biol Med.

[70] Semba RD, Zhang P, Adelnia F, Sun K, Gonzalez-Freire M, Salem N Jr (2019). Low plasma lysophosphatidylcholines are associated with impaired mitochondrial oxidative capacity in adults in the Baltimore Longitudinal Study of Aging. Aging Cell.

[71] Ward-Caviness CK, Xu T, Aspelund T, Thorand B, Montrone C, Meisinger C (2017). Improvement of myocardial infarction risk prediction via inflammation-associated metabolite biomarkers. Heart.

[72] Mapstone M, Cheema AK, Fiandaca MS, Zhong X, Mhyre TR, MacArthur LH (2014). Plasma phospholipids identify antecedent memory impairment in older adults. Nat Med.

[73] Knuplez E, Marsche G (2020). An updated review of pro- and anti-inflammatory properties of plasma lysophosphatidylcholines in the vascular system. Int J Mol Sci.

[74] Adada M, Luberto C, Canals D (2016). Inhibitors of the sphingomyelin cycle: sphingomyelin synthases and sphingomyelinases. Chem Phys Lipids.

[75] Mattson MP, Cutler RG. Sphingomyelin and ceramide in brain aging, neuronal plasticity and neurodegenerative disorders. In: Advances in Cell Aging and Gerontology. Vol 12. Elsevier; 2003. p. 97-115. 10.1016/S1566-3124(03)12006-8.

[76] Katsel P, Li C, Haroutunian V (2007). Gene expression alterations in the sphingolipid metabolism pathways during progression of dementia and Alzheimer’s disease: a shift toward ceramide accumulation at the earliest recognizable stages of Alzheimer’s disease?. Neurochem Res.

[77] Cutler RG, Kelly J, Storie K, Pedersen WA, Tammara A, Hatanpaa K (2004). Involvement of oxidative stress-induced abnormalities in ceramide and cholesterol metabolism in brain aging and Alzheimer’s disease. Proc Natl Acad Sci U S A.

[78] Law BA, Liao X, Moore KS, Southard A, Roddy P, Ji R (2018). Lipotoxic very-long-chain ceramides cause mitochondrial dysfunction, oxidative stress, and cell death in cardiomyocytes. FASEB J.

[79] Monette JS, Gómez LA, Moreau RF, Dunn KC, Butler JA, Finlay LA (2011). (R)-α-Lipoic acid treatment restores ceramide balance in aging rat cardiac mitochondria. Pharmacol Res.

[80] Gao AW, Chatzispyrou IA, Kamble R, Liu YJ, Herzog K, Smith RL (2017). A sensitive mass spectrometry platform identifies metabolic changes of life history traits in C elegans. Sci Rep.

[81] Braun F, Rinschen MM, Bartels V, Frommolt P, Habermann B, Hoeijmakers JH (2016). Altered lipid metabolism in the aging kidney identified by three layered omic analysis. Aging (Albany NY).

[82] Lin L, Cao B, Xu Z, Sui Y, Chen J, Luan Q (2016). In vivo HMRS and lipidomic profiling reveals comprehensive changes of hippocampal metabolism during aging in mice. Biochem Biophys Res Commun.

[83] Park S, Kim BK, Park SK (2021). Supplementation with phosphatidylethanolamine confers anti-oxidant and anti-aging effects via hormesis and reduced insulin/IGF-1-like signaling in C elegans. Mech Ageing Dev.

[84] Zhou L, Ge M, Zhang Y, Wu X, Leng M, Gan C (2022). Centenarians alleviate inflammaging by changing the ratio and secretory phenotypes of T helper 17 and regulatory T cells. Front Pharmacol.

[85] van Dieren JM, Simons-Oosterhuis Y, Raatgeep HC, Lindenbergh-Kortleve DJ, Lambers ME, van der Woude CJ (2011). Anti-inflammatory actions of phosphatidylinositol. Eur J Immunol.

[86] Shi D, Xia X, Cui A, Xiong Z, Yan Y, Luo J (2020). The precursor of PI(3,4,5)P3 alleviates aging by activating daf-18(PTEN) and independent of daf-16. Nat Commun.

[87] Matuoka K, Chen KY, Takenawa T (2003). A positive role of phosphatidylinositol 3-kinase in aging phenotype expression in cultured human diploid fibroblasts. Arch Gerontol Geriatr.

