# Supplementary material for: Serum phospholipids during aging: A comprehensive systematic review of cross-sectional and case-control studies
Source: Health Promot Perspect. 2025 May 6;15(1):23–36. doi: 10.34172/hpp.025.42914 (PMC12125509; doi:10.34172/hpp.025.42914)
Supplement: Supplementary file 1 — The search pattern used for search in PubMed database. [file hpp-15-23-s001.pdf]

Zarezadeh et al, **Health Promotion Perspectives.** 2025;15(1):23-S1
doi: 10.34172/hpp.025.42914
https://hpp.tbzmed.ac.ir

Supplementary file 1. The search pattern used for search in PubMed database

((((((((((((((((((((("ageing"[Title/Abstract]) OR "aging"[Title/Abstract]) OR "healthy ageing"[Title/Abstract]) OR "healthy aging"[Title/Abstract]) OR ("Aging"[Mesh] OR "Healthy Aging"[Mesh]))) OR longevity[Title/Abstract])) OR longevity[MeSH Terms])) OR "Dementia"[Title/Abstract]) OR "cognitive decline"[Title/Abstract])) OR dementia[MeSH Terms]) OR "cognitive dysfunction"[MeSH Terms]))) AND (((((((((((((((((((((("Metabolome"[Mesh]) OR "Metabolomics"[Mesh])) OR "metabonomics"[Title/Abstract]) OR "metabolome"[Title/Abstract]) OR "metabolomics"[Title/Abstract]))) OR lipidome[Title/Abstract]) OR lipidomics[Title/Abstract]) OR sphingolipid*[Title/Abstract]) OR sphingomyelin*[Title/Abstract]) OR phospholipid*[Title/Abstract]) OR phosphatidylcholine*[Title/Abstract]) OR phosphatidylethanolamine*[Title/Abstract]) OR phosphatidylinositol*[Title/Abstract]) OR phosphatidylserine*[Title/Abstract])) OR "plasma lipids"[Title/Abstract])) OR "serum lipids"[Title/Abstract])))) NOT ((((((((((((((((mouse[Title/Abstract]) OR "mice"[Title/Abstract]) OR "rat"[Title/Abstract]) OR "rats"[Title/Abstract]) OR chicken[Title/Abstract]) OR "rabbit"[Title/Abstract]) OR "rabbits"[Title/Abstract]) OR animal[Title/Abstract]) OR "pigs"[Title/Abstract]) OR "broiler"[Title/Abstract]) OR "goats"[Title/Abstract]) OR "hamster"[Title/Abstract]) OR "cats"[Title/Abstract]) OR "dogs"[Title/Abstract])))))
